# *Ostreopsis* cf. *ovata* Bloom in Currais, Brazil: Phylogeny, Toxin Profile and Contamination of Mussels and Marine Plastic Litter

**DOI:** 10.3390/toxins11080446

**Published:** 2019-07-27

**Authors:** Carlos Eduardo J. A. Tibiriçá, Isabel P. Leite, Talita V. V. Batista, Luciano F. Fernandes, Nicolas Chomérat, Fabienne Herve, Philipp Hess, Luiz L. Mafra

**Affiliations:** 1 Centro de Estudos do Mar, Universidade Federal do Paraná, Cx. Postal 61, Pontal do Paraná, PR 83255-976, Brazil; 2 Departamento de Botânica, Universidade Federal do Paraná, Cx. Postal 19031, Curitiba, PR 81531-990, Brazil; 3 LER BO, Station de Biologie Marine, IFREMER, Place de la Croix, F-29900 Concarneau, France; 4 Laboratoire Phycotoxines, IFREMER, Rue de l’Ile d’Yeu, 44311 Nantes, France

**Keywords:** Harmful algal bloom, benthic microalgae, toxic dinoflagellates, ovatoxin, toxin transfer, seafood safety, marine pollution, plastic litter, biofilm formation

## Abstract

*Ostreopsis* cf. *ovata* is a toxic marine benthic dinoflagellate responsible for harmful blooms affecting ecosystem and human health, mostly in the Mediterranean Sea. In this study we report the occurrence of a summer *O.* cf. *ovata* bloom in Currais, a coastal archipelago located on the subtropical Brazilian coast (~25° S). This bloom was very similar to Mediterranean episodes in many aspects: (a) field-sampled and cultivated *O.* cf. *ovata* cells aligned phylogenetically (ITS and LSU regions) along with Mediterranean strains; (b) the bloom occurred at increasing temperature and irradiance, and decreasing wind speed; (c) cell densities reached up to 8.0 × 10^4^ cell cm^−2^ on fiberglass screen and 5.6 × 10^5^ cell g^−1^ fresh weight on seaweeds; (d) and toxin profiles were composed mostly of ovatoxin-a (58%) and ovatoxin-b (32%), up to 35.5 pg PLTX-eq. cell^−1^ in total. Mussels were contaminated during the bloom with unsafe toxin levels (up to 131 µg PLTX-eq. kg^−1^). *Ostreopsis* cells attached to different plastic litter, indicating an alternate route for toxin transfer to marine fauna via ingestion of biofilm-coated plastic debris.

## 1. Introduction

Benthic dinoflagellates belonging to the genus *Ostreopsis* are cosmopolitan, present in both tropical and temperate areas [1]. Several of the eleven *Ostreopsis* species currently described are reported to be toxic, although taxonomic confusion exists as some species were previously described based solely on morphological features, lacking molecular biology analyses [2]. One of the most toxic species, *Ostreopsis* cf. *ovata*, has been responsible for blooms affecting both human and animal health worldwide [3,4,5,6]. This dinoflagellate produces toxins similar to the palytoxins, i.e., isobaric palytoxin (PLTX) and ovatoxins (OVTXs) [7,8], which can intoxicate humans by inhalation or the ingestion of contaminated seafood. However, a great variability in toxin profile among species, strains, and geographic locations exists, leading to different levels of threats to human health [7,9]. Therefore, it is necessary to investigate the molecular identification of the strains in addition to toxicity for assessing the potential risks to human health at each location.

Blooms of *Ostreopsis* spp. are mainly associated with calm waters, rising temperature and high nutrient availability [10,11]. In temperate and subtropical environments, such as the northern Mediterranean coast, blooms occur mostly in summer. In Italy, a multi-step health surveillance system has been put into practice, mainly during the summer, when surveillance programs become pivotal to prevent dangerous exposure of the public to high *Ostreopsis* spp. cell abundances [12]. Interestingly, recurrent intoxication episodes have also been reported during summer among beach users in the northeastern coast of Brazil (Bahia State, ~16° S). Although the effects in humans are similar to those reported during blooms in the Mediterranean, the causative agent of outbreaks in Brazil has not yet been conclusively traced to *Ostreopsis* [13]. Likewise, massive deaths of sea urchins have been reported during periods of high *O.* cf. *ovata* cell abundance on the tropical coast of Brazil (Rio de Janeiro State, ~23° S) [5], but toxin accumulation in marine fauna and potential transfer to human consumers have never been evaluated.

The geographical distribution of *Ostreopsis* spp. and other toxic benthic dinoflagellates has expanded in recent years [14]. It is now suggested that dispersion of epibenthic dinoflagellates in the sea can be facilitated by cell attachment to floating material (i.e., rafts), including seaweeds and marine plastic litter [15,16,17]. Global production of plastic materials has exponentially increased over the last decades, reaching >300 million ton in 2014, and this amount is predicted to be multiplied 6-fold by the year of 2050 [18]. Not surprisingly, plastic pollution has become one of the greatest threats to marine ecosystems, being responsible for adverse environmental effects and even the death of marine organisms, including endangered species [19].

Plastic litter ranges in size from several meters to a few nanometers and can be found in most aquatic ecosystems, including remote marine areas such as deep seas and Antarctic isolated islands [20,21]. Plastic fragments represent up to 92% of the detritus encountered by marine organisms, and may limit their movement, feeding and breathing following entanglement or ingestion [19,22,23,24]. Larger debris (macroplastics, >5 mm) affect mainly big pelagic and benthic marine organisms, such as sea turtles, seabirds, fish and cetaceans [22]. In Brazil, 15–40% of the examined stranded seabirds and 57–100% of sea turtles contained varying quantities of large debris in their stomachs, mostly consisting of plastic fragments [25,26,27]. In addition, smaller particles (microplastics, <5 mm, and nanoplastics, <0.1 µm) currently outnumber larger debris. They can be ingested by or attach to smaller invertebrates and planktonic microorganisms, causing direct negative effects and potentially affecting the entire food chain [28,29,30]. Besides mechanical obstruction, plastic litter can cause intoxication due to the presence of numerous persistent organic pollutants (POPs) and endocrine disruptor chemicals (EDCs) [29]. These chemicals can lead to reproductive disorders or death, increase the risk of disease and modify hormone levels, possibly affecting marine animals and/or zooplankton assemblages [19,28,29]. Furthermore, due to their great surface-to-volume ratio and strong hydrophobic properties, smaller plastic debris can adsorb toxic and persistent organic pollutants, including dichlorodiphenyl trichloroethanes (DDTs), polychlorinated biphenyls (PCBs), polycyclic aromatic hydrocarbons (PAHs) and hexachlorocyclohexanes (HCHs) (reviewed in Lu et al. [31]).

Toxin-producing benthic dinoflagellates can be also found attached to plastics in the sea, including implanted artificial substrate (fiberglass screen; [32]) and floating plastic litter [16]. Approximately 45 species of benthic dinoflagellates are known to produce potent biotoxins, mostly neurotoxins [33] and are potential colonizers of marine plastic debris. It is thus imperative to assess whether highly toxic species, such as *Ostreopsis* spp., can become abundant in plastic litter so that these artificial substrates may act as vectors of biotoxins.

In the present study we report the occurrence of a summer *O.* cf. *ovata* bloom on a coastal island located in the subtropical Brazilian coast (Paraná State, ~25° S). Light, epifluorescence and electron microscopy as well as molecular analysis were carried out for species identification on both field and cultured cells. We examined the vertical and temporal distribution of the bloom and the interaction of *O.* cf. *ovata* with natural and artificial substrates, including different types of plastic litter. The toxin profile was investigated in field-sampled and cultivated cells, as well as in aquatic invertebrates naturally and experimentally exposed to the toxic cells in situ.

## 2. Results

### 2.1. Bloom Detection

**Figure 1:**
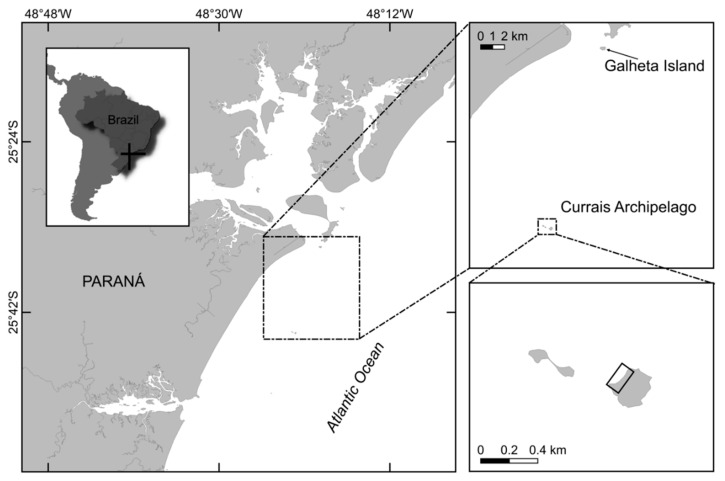
Map of Paraná State Coast (Southwest Atlantic Ocean, Brazil), showing the *Ostreopsis* bloom location (Currais Archipelago, detailed). In the first detailed map an arrow shows the location of Galheta Island, where mussels were firstly collected. In the second detailed map the rectangle shows the exact area affected by the bloom.

**Figure 2:**
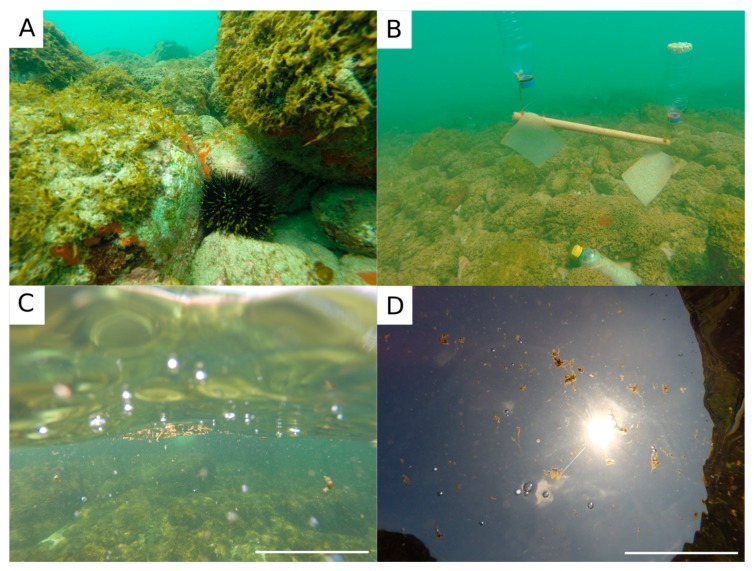
Photographs taken during the *Ostreopsis* cf. *ovata* bloom in Currais Archipelago, southern Brazil: (**A**,**B**) *Ostreopsis* biofilm covering the seafloor; (**C**,**D**) *Ostreopsis* mucous cell aggregates floating at sea surface. Microphytobenthos sampler composed of a fiberglass screen can be seen next to the bottom in “B”. Scale bar (**C**,**D**; focused cell aggregates) = 10 cm.

**Figure 3:**
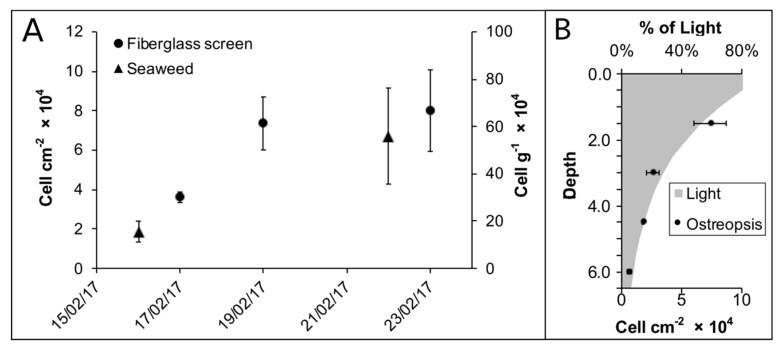
*Ostreopsis* cell densities (**A**) over the sampling period during the 2017 bloom and (**B**) along a vertical profile on February 19th. Light percentage (*L%*) at a given depth (*z*) was calculated by the formula “*L%* = 100% × exp (*−k × z*)”, were “*k*” is the light attenuation coefficient (1.7 divided by the Secchi disc depth) [34].

The bloom was detected by chance during a regular SCUBA dive sampling campaign on February 16th 2017 in Currais Archipelago (Figure 1). Three days later, a yellowish biofilm was noticed covering a ~50,000 m^2^ area of the seafloor, extending from 0 to 8.0 m depth (Figure 2a,b). On February 19th, mucous cell aggregates were found floating abundantly at the sea surface (Figure 2c,d). Cell density over the benthic substrates increased from February 16th to the 19th, remaining similarly high until the last sampling day on February 23^rd^ (Figure 3). *Ostreopsis* reached a maximum of 5.6 × 10^5^ cell g^−1^ of seaweeds fresh weight (fw), and 8.0 × 10^4^ cell cm^−2^ of the artificial substrate-fiberglass screen (Figure 3). Vertical distribution, as assessed on February 19th using artificial substrate, revealed much higher cell densities (7.4 × 10^4^ cell cm^−2^) in shallower areas (1.5 m depth). Cell abundance decreased exponentially as light diminished in deeper areas, reaching 0.6 × 10^4^ cell cm^−2^ at 6.0 m depth (Figure 3). During the 30 days preceding the first sampling campaign, air temperature, sea surface temperature (SST) and solar radiation were increasing, while wind speed and cloud coverage were decreasing. On the first sampling day, daily-average SST had increased from 27.9 to about 28.8 °C, wind had decreased from approximately 6.6 to 4.3 m s^−1^ and surface radiation was ~960 kJ m^−2^. Another bloom recurred in February 2018 at similar environmental conditions: increasing SST, decreasing wind speed and cloud coverage. During both events, samples were obtained for cell culture establishment, however spatio-temporal distribution was only determined for the earlier event, as described above.

### 2.2. Species Identification

**Figure 4:**
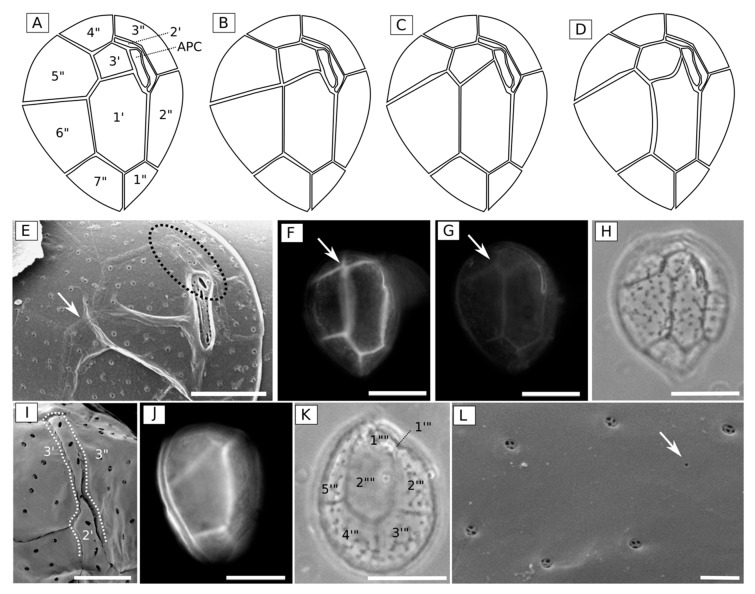
*Ostreopsis* cf. *ovata* from Currais Archipelago, southern Brazil: (**A**–**D**) drawing showing variations in epitheca plate pattern; (**E**,**I**,**L**) scanning electron micrographs (SEM); (**F**,**G**,**J**) epifluorescence micrographs; and (**H**,**K**) phase contrast micrographs. In detail, characteristic features of the taxon: (**E**–**H**) epitheca with variable suture (present, touching in a point, or absent) between 1′ and 5” (arrow); (**E**,**I**) plate 2′separating 3′ from 3” (dotted circle/line); (**J**) a narrow cingulum; (**K**) the epitheca plate pattern; and (**L**) smooth cell surface, with few smaller pores (arrow). Scale bar = 20 µm, except in I (5 µm) and L (1 µm).

Cells from monoclonal cultures (4 strains) and field samples were oval and ventrally slender in apical and antapical views, as assessed by light, epifluorescence and electron microscopy (Figure 4). They were 23.7–65.9 μm (mean = 43.1 μm, standard deviation (SD) = 9.1, *n* = 318) deep (dorso-ventral length, DV), 15.4–48.9 μm (mean = 31.4 μm, SD = 7.1, *n* = 270) wide (W) and 16.7–44.5 μm (mean = 26.1, SD = 5.7, *n* = 45) long (antero-posterior length, AP). The DV/W ratio was 1.04–1.79 (mean = 1.38, SD = 0.15, *n* = 326). Cultivated cells were smaller and more rounded (DV/W ratio = 1.33, SD = 0.13, *n* = 238) than those sampled directly from the field (DV/W ratio = 1.53, SD = 0.12, *n* = 88) (Table 1). All cell dimensions were also more variable in cultures (SD of DV = 8.7, W = 7.5, AP = 6.3) than those sampled from the field (SD of DV = 6.6, W = 5.9, AP = 3.3). The thecal plate pattern was APC 3′ 7′′ 5′′′ and 2′′′′, and the thecal surface was smooth. Mean diameter of thecal pores was 0.28 μm (SD = 0.04, *n* = 6), with internal structures usually splitting it into five poroids, with the presence of a few smaller pores (~0.06 μm in diameter) on the thecal surface (Figure 4L). The first apical plate (1′) was large and hexagonal. Suture of 1′ with the third apical (3′) plate varied from straight to curved in different specimens (Figure 4A–H). The second apical plate (2′) was always narrow and elongated, and located below the APC, reaching the fourth precingular plate (4”), and separating the third precingular (3”) plate from the 3′ plate (Figure 4E,I). In most examined cells, 3′ was pentagonal in shape and contacting 1′, 2′, 3′′, 4′′ and the fifth precingular (5”) plates (Figure 4A,E). However, in some other cells, the suture between 1′and 5” was short or absent. In this case, 3′ was more hexagonal sometimes also touching the sixth precingular (6”) plate (Figure 4B–D,F–H).

Species identification was confirmed by phylogenetic analyses based on ITS region (ITS 1, 5.8S rDNA and ITS 2) and partial LSU rDNA (D8–D10 domains). Both analyses included sequences from five monoclonal cultures, one from a cell pellet obtained from a field sample, and other sequences retrieved from GenBank. The final ITS alignment comprised 51 sequences (including one outgroup sequence) and had a length of 336 base pairs. The best-fit model was found to be GTR + G model (General Time Reversible model) with base frequencies of A = 0.27462, C = 0.18443, G = 0.19523, T = 0.34572, assuming a gamma distribution shape (G = 0.583). For LSU D8–D10, the final alignment comprised 37 sequences and had a length of 817 base pairs, and the best-fit model was found to be GTR +I+ G model, with base frequencies of A = 0.28459, C = 0.17103, G = 0.25154, T = 0.29284, assuming invariable sites (I = 0.557) and gamma distribution shape (G = 0.523).

**Figure 5:**
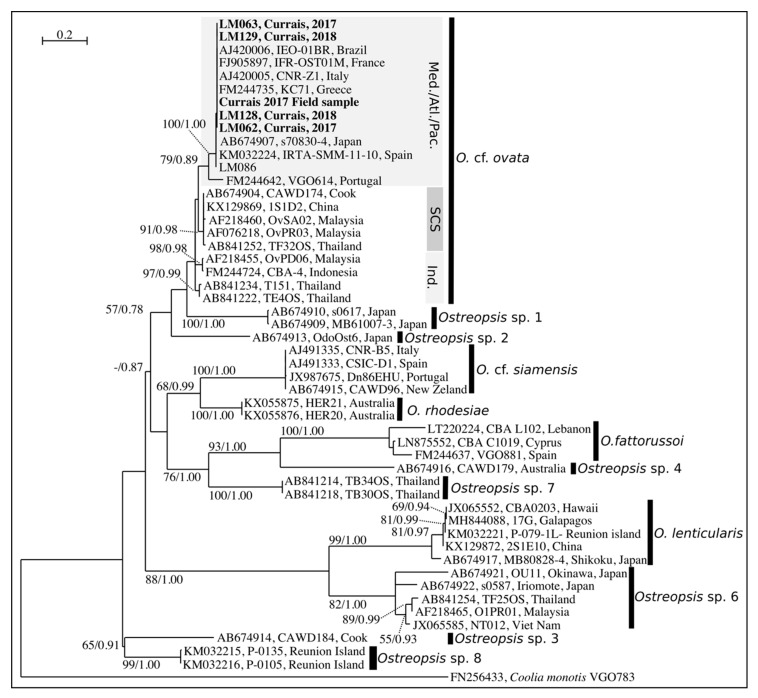
Maximum Likelihood phylogenetic tree inferred from ITS 1, 5.8S and ITS 2 sequences of various *Ostreopsis* strains. Currais field sample and monoclonal cultures are indicated by bold face and a gray background. *Coolia monotis* is used as an outgroup. Black vertical bars show distinct *Ostreopsis* clades. For *O.* cf. *ovata*, three subclades are shown: “Med./Atl./Pac.” for Mediterranean, Atlantic and Pacific subclade; “SCS” for the South China Sea subclade and “Ind.” for the Indian ocean and Thailand subclade. Numbers at nodes represent bootstrap support values from Maximum Likelihood (ML) and posterior probabilities from Bayesian Inference (BI).

**Figure 6:**
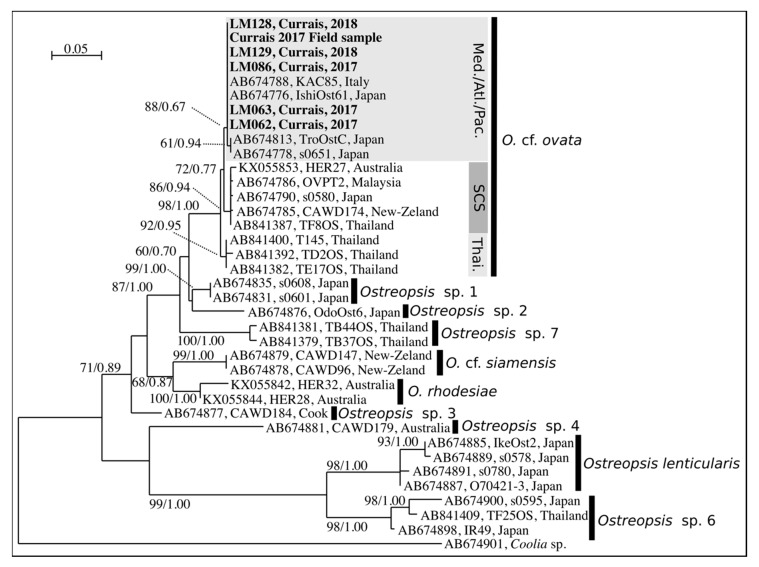
Maximum Likelihood phylogenetic tree inferred from LSU D8–D10 sequences of various *Ostreopsis* strains. Currais field sample and monoclonal cultures are indicated by bold face and a gray background. *Coolia* sp. is used as outgroup. Black vertical bars show distinct *Ostreopsis* clades. For *O.* cf. *ovata*, three subclades are shown: “Med./Atl./Pac.” for Mediterranean-Atlantic-Pacific, “SCS” for South China Sea, and “Thai.” for Thailand subclade. Numbers at nodes represent bootstrap support values from Maximum Likelihood (ML) and posterior probabilities from Bayesian Inference (BI).

Phylogenetic analyses were performed with two methods of reconstruction: maximum likeligood (ML) and Bayesian Inference (BI,). Considering that ML and BI analyses gave the same tree topology and relationships among clades, only the majority-rule consensus tree of the ML analysis is shown. Twelve distinct clades were found in the phylogeny inferred from ITS sequences (*O.* cf. *ovata*, *O.* cf. *siamensis*, *O. rhodesiae*, *O. fattorussoi*, *Ostreopsis* spp. 1–4 clades, *O. lenticularis*, *Ostreopsis* spp. 6–8) and ten clades from LSU D8–D10 (no sequences were available for *O. fattorussoi* and *Ostreopsis* sp. 8 (Figure 5 and Figure 6). All sequences from Currais Archipelago (5 monoclonal cultures and cells from the field sample) clustered within the Mediterranean subclade of *O.* cf. *ovata* (Figure 5 and Figure 6).

### 2.3. Colonization of Plastic Litter by Microalgae

**Figure 7:**
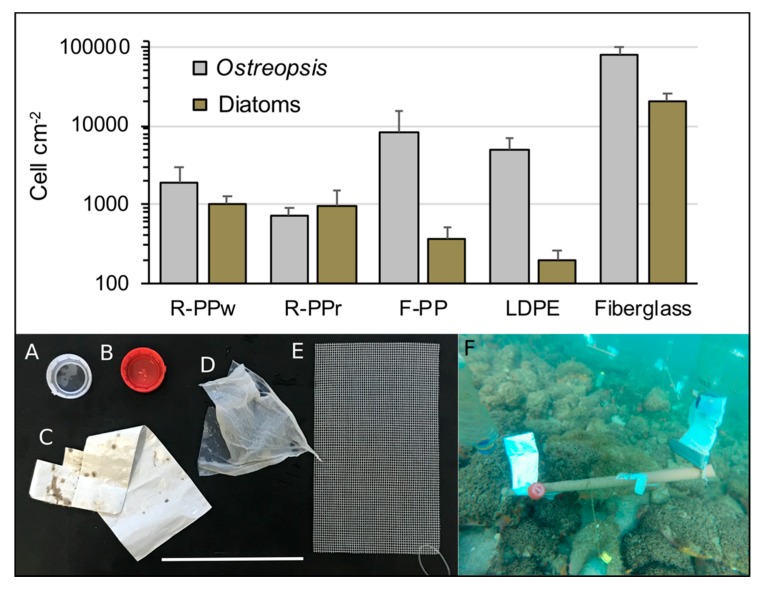
Cell densitiy (cell cm^-2^, log scale) of *Ostreopsis* and co-occurring diatoms on the following plastic materials: (**A**) white and (**B**) red rigid polypropylene bottle cap (R-PPw and R-PPr, respectively), (**C**) flexible polypropylene plastic packaging (F-PP), (**D**) flexible, low-density polyethylene plastic bag (LDPE), and (**E**) fiberglass screen (Fiberglass) following (**F**) 24 h of exposure in Currais Archipelago seawater (6.0 m depth). Scale bar = 10 cm (**A**–**E**).

During the 2017 bloom, the abundance of attached *Ostreopsis* cells varied among artificial samplers made of different materials, including a fiberglass screen similar to that described in Tester et al. [32], and four types of plastic litters commonly found in the gastro-intestinal tract of green-turtles in the region [26,35]. Cell density on rigid polypropylene bottle caps (R-PP) was lower (mean 1.3 × 10^3^ cell cm^−2^), with no significant differences between white (R-PPw) or red (R-PPr) colors (Figure 7). Sections of low density polyethylene plastic bags (LDPE) accumulated up to 4.9 × 10^3^ cell cm^−2^, while higher densities were found on those made of flexible polypropylene plastic packing (F-PP) (mean 8.4 × 10^3^ cell.cm^−2^) and fiberglass screen (mean 80 × 10^3^ cell cm^−2^). Cell density of co-occurring diatoms was much higher in R-PP (mean = 9.9 × 10^2^ cell cm^−2^, or 76% of *Ostreopsis* cell abundance) compared to both flexible plastic litter—LDPE and F-PP (mean = 2.8 × 10^2^ cell cm^−2^, only 4% of *Ostreopsis* cell abundance). Proportionally, cell density of diatoms was also lower than that of *Ostreopsis* on fiberglass screens (26% of *Ostreopsis* cell abundance) after 24 h of exposure (Figure 7).

### 2.4. Toxin Production and Accumulation in Marine Organisms

**Figure 8:**
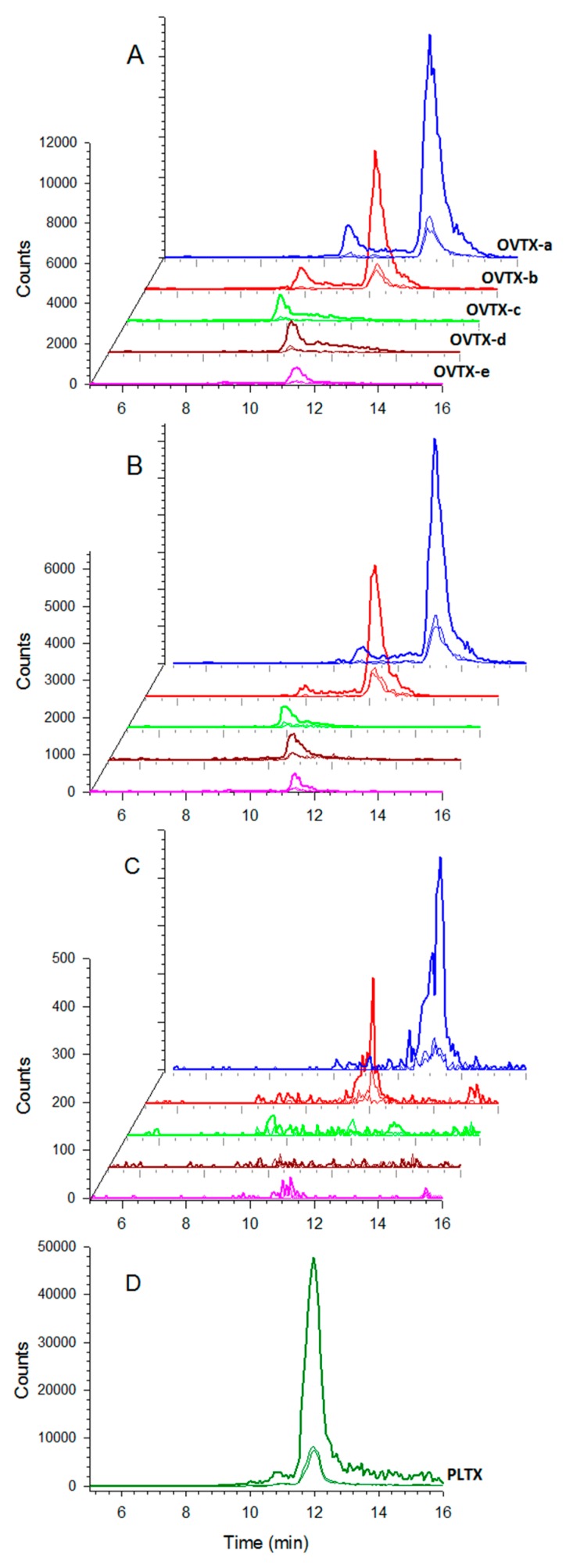
Multiple reaction monitoring (MRM, positive ionization mode) LC-MS/MS chromatogram of ovatoxin (OVTX)-a (*m*/*z* 1324.2→327.2; 1315.2→327.2; 877.2→327.2), OVTX-b (*m*/*z* 1346.3→371.2; 1337.3→371.2; 891.8→327.2), OVTX-c (*m*/*z* 1354.3→371.2; 1345.3→371.2; 897.2→327.2), OVTX-d (*m*/*z* 1332.2→327.2; 1323.2→327.2; 882.5→327.2), OVTX-e (*m*/*z* 1332.2→343.2; 1323.2→343.2; 882.5→343.2) and palytoxin (PLTX) (*m*/*z* 1340.2→327.2; 1331.2→327.2; 887.8→327.2) in selected samples of (**A**) *Ostreopsis* cf. *ovata* monoclonal culture; (**B**) cell pellet from the 2017 *O.* cf. *ovata* bloom in Currais Archipelago; (**C**) *Perna perna* mussel whole tissue homogenate, and (**D**) PLTX standard. See Methods for details.

**Figure 9:**
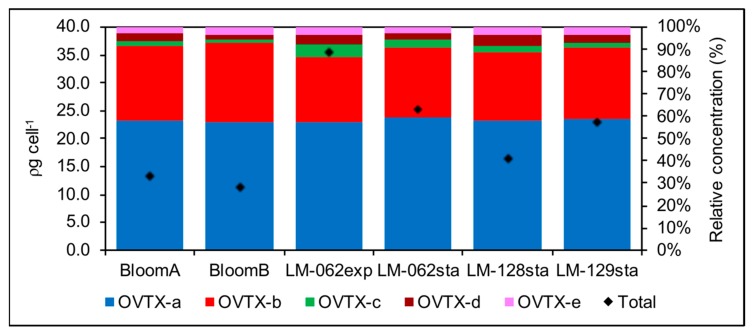
Intracellular toxin content and toxin profile of *Ostreopsis* cf. *ovata* cells collected directly from the 2017 bloom in Currais Archipelago (two replicates: “BloomA” and “BloomB”), or obtained from monoclonal cultures sampled at either exponential (exp) or stationary growth phase (sta). Strain LM-062 was established from a sample collected during the 2017 bloom, and strains LM-128 and LM129 from a second bloom in the same place, in February 2018.

The presence of Ovatoxin (OVTX) -a, b, c, d and e was detected in all samples, including cell pellets from the bloom and cultured cells (Figure 8). The total toxin quota was higher in cultures (up to 35.5 pg cell^−1^ in the stationary phase of LM-062 culture) than in field samples (mean of 12.2 pg cell^−1^) (Figure 9). Intracellular toxin levels also varied among different strains at equivalent growth stage. Strains isolated from the 2018 bloom (LM-128 and LM-129) were slightly less toxic than those previously established (Figure 9). The toxin profile, however, was more conservative, composed mostly of OVTX-a (approx. 58% of the total toxin content in all samples) and OVTX-b (30–35% depending on the sample). Overall, OVTX-c, OVTX-d and OVTX-e contributed each to approximate 3% of the total toxin content, except at exponential growth phase of culture LM-062, in which OVTX-c and OVTX-d represented up to 5% of the total toxin content each (Figure 9). Isobaric palytoxin was not detected in any sample (Figure 8).

During the 2017 bloom (on February 22rd), we detected toxin transfer to *Perna perna* mussels that were collected on a nearby (~16.5 Km) location—Galheta Island—and transplanted to the bloom area in Currais Arquipelago, remaining at the bottom (1.5 m depth) for 24 h. Up to 131 µg kg^−1^ of total OVTX (mean 98.0 µg kg^−1^, *n* = 5) were accumulated in mussels after the 24 h exposure period (Table 2). In general, toxins in mussels were mostly composed of OVTX-a (68.5%) and OVTX-b (27%), with smaller amounts of OVTX-c (up to 10%) and OVTX-e (up to 4.5%) (Table 2). Curiously, mussels sampled from Galheta Island already contained smaller OVTX amounts (up to 32.9 µg kg^−1^), even though *Ostreopsis* cell abundance were much lower there (up to 5.0 × 10^3^ cell g^−1^ of seaweeds fw) compared to the bloom area in Currais (up to 560 × 10^3^ cell g^−1^ of seaweeds fw). In addition, OVTX-a and OVTX-b (29.5 µg kg^−1^ in total) were also found in a single sample of coral (*Palythoa* sp.) naturally occurring in Currais. Conversely, toxins were undetectable (<20 ng PLTX-eq. mL^−1^) in sea urchins (*Echinometra lucunter*) and one sea cucumber (*Holothuria grisea*) sampled in the same location (Table 2).

## 3. Discussion

### 3.1. Taxonomy and Phylogeny of Ostreopsis Species: Difficulties in Identifying the Toxic Bloom-Forming O. cf. ovata

From the eleven species of *Ostreopsis* described (namely *O. siamensis*, *O. lenticularis*, *O. ovata*, *O. heptagona*, *O. mascarenensis*, *O. labens*, *O. belizeana*, *O. caribbeana*, *O. marina*, *O. fattorussoi* and *O. rhodesiae*), only the two most recent descriptions included genetic data (*O. fattorussoi* and *O. rhodesiae*) [2,36,37]. Moreover, *Ostreopsis* spp. exhibit great intra-specific variability and inter-specific similarity in cell morphology, leading to significant problems regarding the taxonomy of the genus [2,38,39]. Recently, some clarification was obtained on oval-shaped larger-celled Ostreopsis species, with the re-description of *O. lenticularis* from its type locality and the indication that *Ostreopsis sp*. 6 may correspond to the originally described *O. siamensis* [2]. However, for the smaller-celled species (*O.* cf. *ovata*, *O.* cf. *siamensis* and similar species), the confusion regarding distinctive morphological features continues.

Cell shape and size was originally noted as a distinguishing feature for *Ostreopsis* cf. *ovata* identification [40], but the cell size of *Ostreopsis* cf. *ovata* and other smaller-celled species may exhibit great variability and overlap, as shown in the Mediterranean Sea and Atlantic Iberian coast [38,39]. This variability was also observed in the present study in Brazil, in which cells from the same strain varied up to 2-fold in cell length (Table 1). Also, our cultivated cells exhibited different morphology (i.e., smaller size and more rounded) when compared to field samples, as previously reported [38]. This could be the effect of either high nutrient content in the culture media [38] or differences in cell stages [41], and emphasizes that descriptions from cultures should be interpreted cautiously. However, despite the concerns of using cell shape in *Ostreopsis* taxonomy, it is important to point out that *O.* cf. *ovata* cells sampled from Currais appeared wider than those originally described as *O. ovata* [40].

As size and shape overlap among species, plate characteristics were used as a distinguishing morphological feature in recent *Ostreopsis* descriptions [37,42]. However, even that feature was shown to be quite variable in the present study, and was not sufficient to separate *O.* cf. *ovata* from *O. fattorussoi* and *O. rhodesiae*: (a) for *O. fattorussoi* the presence of a curved suture between plates 1′and 3′, making plate 3′ look hexagonal, was reported as a distinguishing characteristic [36], but in the present study (Figure 4H) it was observed that 1′/3′ curvature can be variable and should not be used as a sole characteristic; (b) for both *O. rhodesiae* and *O. fattorussoi*, the elongated second apical plate (2′) separating the third apical plate (3′) and the third pre-cingular plate (3”) has been proposed as a distinguishing feature [36,37], however, this was also observed in our *O.* cf. *ovata* cells (Figure 4). Curiously, the 2′ plate that was originally described to be short and limited to the apical pore length (not separating 3′ from 3”) in *O. ovata* (see drawings by Fukuyo [40]), have proved to be elongated in most recent pictures of cells belonging to the phylogenetic clade named as *O.* cf. *ovata* (see Figure 4A,E,I in the present study; Figure 4C,J in Penna et al. [39]; Figures 11 and 12 in Zhang et al. [43]; and Figure 55C in Hoppenrath et al. [33]).

The clade named as “*O.* cf. *ovata*” includes at least three morphologically identical but genetically distinct morphotypes. Since Sato et al. [44] found all three morphotypes in the *O. ovata* type locality (Ryukyu Islands, Japan [40]), it is not possible to associate either one with this taxonomic designation (cryptic diversity). Therefore, this species should be considered a species complex until further clarification. In the subclade of the *O.* cf. *ovata* species complex where the sequences from Currais aligned it is possible to find at least two strains from the Mediterranean Sea (IFR-OST01M and KC71, Figure 5) that were previously reported to be toxic [45].

### 3.2. Bloom Formation, Toxin Production and Contamination of Marine Organisms

In Brazil, *Ostreopsis* blooms have been previously reported in the oceanic archipelago of São Pedro e São Paulo (up to 9.9 × 10^4^ cell g^−1^ of seaweeds [46]) and the coast of Rio de Janeiro State (up to 1.5 × 10^5^ cell g^−1^ [47]), where negative effects to marine invertebrates have been documented [5]. The bloom described herein, however, is the first report for subtropical Brazilian waters. Cell abundances during the 2017 summer bloom in Currais Archipelago (5.6 × 10^5^ cell g^−1^ of seaweeds or 8.0 × 10^4^ cell cm^-2^ on artificial substrates) were equivalent to those reported in the Mediterranean (e.g., [48]), where extensive *O.* cf. *ovata* blooms are frequent and cell abundances of up to 7.2 × 10^6^ cell g^−1^ of seaweeds or 6.4 × 10^4^ cell cm^−2^ on artificial substrates can be reached [45,48,49]. Those massive blooms have often been associated with negative impacts to marine organisms and human health, due to the toxins produced by *Ostreopsis* [3,50,51]. Considering that cells from southern Brazil contained comparable toxin amounts, negative effects to marine fauna and human health are expected.

The 2017 *O.* cf. *ovata* bloom in Currais occurred after a period of increasing light availability. Additionally, much higher cell abundances were observed at shallower depths, suggesting that increased light intensity is an important factor triggering *O.* cf. *ovata* blooms. However, despite similar observations in previous field studies [52], laboratory experiments reported controversial results. Overall, the general environmental conditions preceding the bloom in Currais (i.e., warmer water temperatures and lower turbulence) were similar to those experienced in the Mediterranean Sea prior to *O.* cf. *ovata* blooms [10]. One year later, in February 2018 (austral summer), another *O.* cf. *ovata* bloom coincided with the period of maximum annual water temperatures (>28 °C) in Currais Archipelago; similar to what has been continuously observed in the Mediterranean (>25 °C [10]). Noteworthy, periods of high irradiance, warm temperature and low water turbulence only occur in the southern West Atlantic Ocean during short periods of the year in mid-summer and early autumn. Our results indicate that *O.* cf. *ovata* blooms should be carefully monitored over the subtropical Brazilian coast.

The southern Brazilian *Ostreopsis* populations sampled herein have not only the same genotype and similar environmental requirements for bloom formation, but also exhibit similar capacity to produce toxins as those causing toxic events in the Mediterranean Sea. The toxin profile in our cultures and field-sampled cells was compared to those registered in regions where harmful blooms are frequent (i.e., mainly ovatoxin-a and -b) (Table 3). Similarly, toxin contents (i.e., total cell quota) in *O.* cf. *ovata* cells from Currais (up to 35.5 pg cell^−1^) are in the same range as those reported for the northeastern Brazilian coast (21.0–43.4 pg cell^−1^) and most strains isolated from Europe (6.0–75.0 pg cell^−1^). Sporadically, OVTX cellular quotas can reach much higher values in *O.* cf. *ovata*, as reported in Spain (250 pg cell^−1^), French Mediterranean (300 pg cell^−1^) and in southeastern Brazil (468 pg cell^−1^) (Table 3). Thus, the risks for negative impacts of *O.* cf. *ovata* blooms to marine fauna and human health should be continuously monitored in southern Brazil.

Humans and domestic animals can be intoxicated by *Ostreopsis* toxins upon contact with toxin-containing aerosol on the beach, as commonly documented in the Mediterranean [12,50,51,53] and suggested in the northeastern coast of Brazil [13]. Palytoxin is considered one of the most toxic naturally occurring non-peptide compounds via oral exposition, and cases of human death related to the ingestion PLTX-contaminated seafood have been reported [53,54]. In laboratory studies with marine organisms, *O.* cf. *ovata* cells exhibited acute toxicity to sea urchin gametes and larvae [55], as well as larval stages of crustaceans—*Artemia salina* brine shrimps, *Tigriopus fulvus* copepods and *Amphibalanus amphitrite* barnacles—and juvenile fish, *Dicentrarchus labrax* [3,56]. Moreover, toxins from *O.* cf. *ovata* are likely involved in massive deaths of adult sea urchins during natural blooms, as reported in New Zealand [4] and southeast Brazil [5].

There exists no current regulatory limit for PTX-like compounds in seafood [57], however, a 30 µg kg^−1^ safety level in seafood is recommended in Europe [58], where accumulation of these toxins has been reported in sea urchins and bivalve mollusks during an *O.* cf. *ovata* bloom [45]. In the present study, no commercial bivalve species were found in the area affected by the bloom in Currais Archipelago. We thus decided to collect commercial-sized mussels (*Perna perna*) from the nearby Galheta Island, a place ~16.5 km distant from the bloom area and near the shore (Figure 1), where people occasionally go to collect mussels as a food source. We left some individuals in Currais for 24 h to investigate the potential accumulation of PLTX-like compounds from *Ostreopsis* cells, and examined others for the presence of toxins. Surprisingly, the mussels were already contaminated prior to transplantation, containing up to 32.9 µg PLTX-eq. kg^−1^ (average 22.3 µg kg^−1^), even though *O.* cf. *ovata* cell densities were more than 100-fold lower in Galheta Island. After 24 h of exposure to higher *O.* cf. *ovata* cell densities at the bloom area in Currais Archipelago, toxin concentrations reached up to 130 µg kg^−1^ in transplanted mussels. These values are within the same order of magnitude as the toxin concentration values found in mussels (up to 217 µg.kg^−1^) during an *O.* cf. *ovata* bloom on the French Mediterranean coast [45]. Considering the short exposure time of *P. perna* mussels in the present study and the relatively high toxin concentrations accumulated, these organisms may be considered potential intoxication vectors to humans and can be used as a sentinel for the presence of this toxin in coastal marine ecosystems. The risks for cases of human intoxication by *Ostreopsis* toxins in this region should be considered by local authorities engaged in seafood safety programs.

Toxins of *Ostreopsis* can accumulate at lower levels in several marine organisms other than bivalves, including fishes, crustaceans, cephalopods, gastropods, echinoderms and sponges [45,63,64]. In the present study, we were not able to detect toxins in sea urchins nor in a single sea cucumber individual. Even though the animals were in close association with the *Ostreopsis* biofilm at the bottom of Currais Archipelago, we examined entire animals (as whole tissue homogenates), and this procedure may have diluted any toxin amount possibly present in specific tissues of these animals. Conversely, we were able to detect and quantify OVTX-a (20.0 µg kg^−1^) and -b (9.5 µg kg^−1^) in a single specimen of coral (*Palythoa* sp.), although it was not possible to determine whether the toxin had been assimilated by the coral or contained in *Ostreopsis* cells attached to the coral surface and pores. Toxin values in coral were similar to those reported in non-bivalve invertebrates during *O.* cf. *ovata* blooms in the Mediterranean [64,65].

### 3.3. The Plastic Litter Problem

In the ocean, plastic debris can be readily covered by a biofilm composed of bacteria and benthic microalgae, mostly diatoms [66,67,68]. Dinoflagellates, including *Ostreopsis*, can also attach their cells to plastic litter, but in general with less adhesion capacity [16,17]. In the present study, toxin-producing dinoflagellates were dominant over diatoms in plastic litter left in the water for 24 h during an *O.* cf. *ovata* bloom. Thus, the role of toxic cell-coated plastic debris as artificial toxin vectors for marine fauna, as well as the interactive harmful effects elicited upon their ingestion, must be thoroughly considered and examined.

The process of plastic colonization is not only dependent on the microorganisms present in the environment, but also on the plastic characteristics and the position of the plastic litter in the water column [66]. In our study, *O.* cf. *ovata* attached more abundantly to more flexible plastic materials, probably due to the movement of the plastic in the water facilitating “capture” of floating *Ostreopsis* cells that detach from substrate in mucous aggregates. The abundance of *Ostreopsis* was one order of magnitude higher on fiberglass screen, showing that its design is very efficient for sampling benthic dinoflagellates—probably due to its higher surface/volume ratio and the flexibility associated with the rough surface of the fiberglass filaments. The cell abundance of *Ostreopsis* was lower on rigid plastics, in which diatoms were present at equivalent numbers.

*O.* cf. *ovata* produces large quantities of mucus in static cultures, and cells aggregate into mucus strings. In the field, favored by the action of waves or currents on the sea floor, mucous *Ostreopsis* cell aggregates detach from the bottom and float. On their way to the surface, these sticky aggregates may come into contact with plastic litter, allowing its colonization by epibenthic *Ostreopsis* cells. Plastic debris (or other rafts) covered by toxic cells may thus become an alternate route for toxin transfer from benthic *O.* cf. *ovata* bloom to pelagic organisms.

Ingestion of plastic litter is common among sea turtles, seabirds, marine mammals and fish [22]. Apart from the fiberglass screen, included here for comparative purposes, the plastic materials tested in the present study (i.e., packing plastic, plastic bags and plastic bottle caps) are among the most abundant and common litter types in the stomachs of sea turtles found dead-stranded over the Brazilian coast (up to 82% of examined individuals) [26,69,70]. In the worst case, ingestion of large amounts of plastic litters can be responsible for the death of sea turtles due to suffocation or obstruction of their digestive systems [70]. Harmful effects of plastic ingestion are expected to be exacerbated in case plastic debris contain adsorbed toxic substances, such as persistent organic pollutants (reviewed in Lu et al. [31]). Likewise, marine plastic litters may be covered by moderate to large amounts of toxic micro-algal cells, as demonstrated for *O.* cf. *ovata* herein (up to 4900 cells cm^−2^) and—to a lesser extent—in another recent study in the Mediterranean Sea (up to 260 cells cm^−2^ [16]). The presence of abundant *O* cf. *ovata* cells covering plastic litters that drift around in sea turtle feeding grounds like Currais [71] is disturbing. Neurotoxins produced by *O.* cf. *ovata* can be highly toxic to marine animals (e.g., [14]), and plastic litter may contain high toxin doses during *Ostreopsis* bloom, as indicated here. For instance, a single 100-cm^2^ (10 × 10 cm) low density polyethylene fragment was found to contain 8 × 10^5^ cells of *O* cf. *ovata* producing 12 pg cell^−1^ of PLTX-like compounds, thus representing a dose of ~10 ug of PLTX-like compounds. The possibility of chronic and acute intoxication of sea turtles (and other animals that ingest plastic litter) due to the ingestion of toxic microalgae-containing plastic litter should be therefore considered.

## 4. Conclusions

*Ostreopsis* cf. *ovata* is a toxic marine benthic dinoflagellate usually responsible for harmful bloom events in the Mediterrenean Sea. In the Currais Archipelago, southern Brazil, this species formed a dense bloom with similar visual characteristics: a yellowish biofilm covering an extensive area of the seafloor, and the appearance of floating mucous cell aggregates. The bloom occurred following a period dominated by similar weather conditions (increasing temperature and decreasing turbulence) to those triggering Europeans events. Parallel morphological and phylogenetic analyses indicated that *O.* cf. *ovata* cells occurring in this part of the western Atlantic Ocean belong to the same genotype as the Mediterranean bloom-forming populations. Toxin intracellular quotas and profile were also equivalent to those found in Europe, suggesting the risks for harmful effects to marine fauna and human health in this area. Moderate toxin concentrations were found in edible mussels during the bloom. Cell densities were equally high on both natural and artificial substrates. Cells of *O.* cf. *ovata* attached to different types of plastic litters that are commonly ingested by sea turtles in this area. The ingestion of biotoxin-coated plastics by these and other animals may thus cause other health damages besides suffocation or obstruction of their digestive tracts.

## 5. Materials and Methods

### 5.1. Sampling

Sampling was conducted from February 16th to 23rd 2017, on a site located in Currais Archipelago (25° 44′ 06.75” S, 48° 22′ 01.89” W), a set of three small islands on the Brazilian subtropical coast (Figure 1). Samples of seaweeds (*n* = 12) and artificial substrates (*n* = 20; Figure 2B) were collected and processed following the procedure described in Tester et al. [32]. The artificial substrate consisted of rectangular pieces of fiberglass screens (10 × 15 cm; ~2 mm mesh size; 174 cm^2^ surface area; Figure 7E). Substrates were positioned in triplicate about 30 cm above the seafloor with the aid of small floats (Figure 2B), and maintained for 24 h. Samples were vigorously shaken to detach particles from the seaweeds or artificial substrates, and the seawater containing *Ostreopsis* cells was divided in three aliquots of 200 mL each: (a) one was used for observation and isolation of living cells; the second one was concentrated by centrifugation to obtain cell pellets for toxin analysis; and (c) the last one was fixed with 1% lugol iodine solution for microscopic counting (1 mL aliquots in triplicate) on a Sedgewick-Rafter chamber.

On February 22nd, a field experiment was conducted with different plastic litters similar to those most commonly found in sea turtle stomachs in the region [26,35]. The plastic items (*n* = 16, four of each type; Figure 7) were installed in the field and processed in the same way as the artificial substrates described above, also remaining in the water ~30 cm above the seafloor for 24 h (Figure 7F). Plastic litters used included rigid polypropylene (R-PP) bottle caps of white and red colors, and sections of flexible polypropylene (F-PP) plastic packing and flexible, low-density polyethylene (LDPE) plastic bags (Figure 7).

### 5.2. Cultures

Cells of *Ostreopsis* were isolated using a capillary pipette following successive washing in sterile, local filtered seawater. After initial growth through consecutive cell divisions, the volume of culture was successively doubled by transferring the old aliquot to a larger microplate well containing an equivalent volume of sterile diluted f/2 media (f/4), without silica and ~32 salinity. From 10 mL wells, cultures were transferred to 50 mL and then to 250 mL Erlenmeyer flasks, where they were maintained at 26 °C under a 12:12 h light cycle (irradiance of 70 ± 20 μmol m^−2^ s^−1^). For toxin analysis, cultivated cells (exponential and stationary growth phase) and field samples (100–200 mL) were harvested by centrifugation (2332× *g*, 5 min), the supernatant was removed, and samples were stored at −20 °C. Prior to toxin analysis, the frozen pellets were lyophilized.

### 5.3. Morphological Observations

Species identification was based mainly on original and recent *Ostreopsis* spp. descriptions (e.g., [36,37,40]). Cell size was measured from photomicrographs using the image-processing software (AxioVision^®^ LE, Zeiss^®^, Oberkochen, Germany). Pictures were taken under 200× magnification using a digital camera (AxioCam^®^ ERc 5s, Zeiss^®^, Germany) coupled to an inverted light microscope (Vert.A1, Zeiss^®^, Oberkochen, Germany). Thecal plate tabulation (following Hoppenrath et al. [33]) was examined under epifluorescence microscopy (BX51, Olympus^®^, Tokyo, Japan) after adding a small drop of calcofluor white to *Ostreopsis* samples mounted on a glass slide. Additionally, cells were stressed to promote ecdysis [72], by adding a few drops of sodium thiosulfate on live *Ostreopsis* samples, and plates were observed under phase-contrast inverted microscopy.

Prior to electron microscopy (SEM) observations, bloom samples were preserved with neutral iodine lugol solution (1%), and cultured *Ostreopsis* cells with neutral and acidic lugol (1%) and glutaraldehyde solutions (5%). Small aliquots of the samples (2–5 mL) were placed on a 5-µm Millipore filter or on a 20-µm plankton net, rinsed with distilled water, and dehydrated in a series of increasing ethanol concentrations (30%, 50%, 70%, 90%, 95% and 100%), followed by critical point drying. Samples were finally mounted on a stub and sputter coated with gold palladium. Cells were observed using a JEOL^®^ JSM 6360-LV (Japan) microscope at 15 Kv.

### 5.4. DNA Amplification, Sequencing and Molecular Phylogeny

Cultivated cells and field samples (10 mL) were harvested by centrifugation (2332× *g*, 5 min), the supernatant was removed and replaced by ethanol to preserve the samples until the DNA analysis. Before the amplification, single cells from the ethanol-preserved samples were isolated with a glass capillary and washed six times with deionized water. Single *Ostreopsis* cells were placed in PCR tubes (at least two tubes for each sample) with 1–3 µL of deionized water and stored at −20 °C before the direct PCR amplifications.

Two consecutive PCR reactions (nested PCR) were performed to amplify the rDNA regions ITS1-5.8S-ITS2 (ITS) and LSU (D8–D10). For the first PCR reaction, 2.5 µL of each primer (ITSfw and OSTD10R, Table 4), 12.5 µL of PCR Master Mix 2X (Promega, Madison^®^, WI, USA) containing the Taq DNA polymerase, dNTPs, MgCl_2_ and reaction buffers, and 6.5 µL of nuclease free water were added to the tube. The PCR were performed in a Biometra TOne, thermocycler (Analytik Jena) as follows: one initial denaturation step at 95 °C for 2 min, followed by 35 cycles at 95 °C for 30 s, 50 °C (melting temperature, “MT”) for 1 min, and 72 °C for 1 min, and a final elongation at 72 °C for 5 min. For the second PCR reaction, 1 µL of the first product were added to a new tube containing 2.5 µL of each primer (ITSfw and D3B for ITS region; D8 and OSTD10R for D8–D10; Table 4), 12.5 µL of GoTaq^®^ G2 Hot Start Green Master Mix (Promega^®^, Madison, WI, USA) and 6.5 µL of nuclease free water. The second PCR was performed as the first, chaging the MT to 62 °C for ITS region, and 47 °C for D8–D10. DNA amplifications were controlled by electrophoresis on agarose gel. Positive samples were purified and sequenced as described in Moreira-Gonzalez et al. [73].

The alignment and phylogenetic analyses were performed as described in Chomérat et al. [2], with the following modifications: both ITS and D8–D10 rDNA region datasets were aligned using MAFFT algorithm with selection of the q-ins-i strategy; poorly aligned positions were re-moved using Gblocks algorithm; the most appropriate model of sequence evolution was selected using jModeltest2 v. 2.1.10; GTR+I+G and GTR+G were the model used for Maximum Likelihood (ML) and Bayesian Inference (BI) analysis of the D8–D10 and ITS regions, respectively; 2,000,000 generations were used in BI analysis for both alignments, with sampling every 100 generations; the posterior probabilities of each clade were calculated from the remaining 20,000 trees.

### 5.5. Sampling and Processing of Marine Fauna

In order to evaluate toxin uptake during the *Ostreopsis* bloom, sea-urchin individuals (*n* = 4), a pool of coral polyps and one sea cucumber individual were opportunistically sampled by snorkeling from the affected area in Currais Archipelago. Additionally, ten mussels (8–11 cm long) were collected on a nearby location in Galheta Island (distant ~16.5 km from Currais and ~2.5 km from the shore; 25°35′7.84” S, 48°19′17.92” W) and five of them were transplanted to the bottom of the area affected by the bloom in Currais, where they remained for 24 h before sampling. The other five individuals were immediately transported to the laboratory. All animals were promptly triturated using a tissue homogenizer (T 10 basic ULTRA-TURRAX^®^, IKA, Staufen, Germany), and the homogenates were extracted in methanol (HPLC grade, Merck^®^, Darmstadt, Germany) at a 9:1 (*v*:*v*) ratio, followed by sonication (130 W, CPX130, Cole Parmer^®^, Vernon Hills, IL, USA) during 3 min with pulses of 3 s and intervals of 1 s, at 80% amplitude. Extracted samples were centrifuged at 2332× *g* for 5 min, filtered with syringe filters (PVDE, 0.22 µm, Analitica^®^, São Paulo, Brazil) and kept frozen until the toxin analysis.

### 5.6. Toxin Analysis

Prior to toxin analysis, cell pellets (from cultures or field samples) were sonicated in bath ultrasound (Transonic TI-H-15, Elma^®^, Wetzikon, Switzerland) at 45 kHz for 15 min with a methanol/water (9:1, *v*/*v*) solution. The mixture was centrifuged at 1200× *g* for 15 min, and the supernatant was passed through a centrifuge NanoSep filter (0.2 µm Nylon, PALL^®^, Portsmouth, UK) and recovered into plastic vials with conical insert. Extracts from marine fauna were concentrated 10-fold by evaporating 1-mL aliquots with nitrogen gas at 40 °C, followed by re-suspension in 0.1 mL MeOH 90%.

Filtered extracts from both cell pellets and marine organism samples were analyzed by liquid chromatography coupled to tandem mass spectrometry (LC-MS/MS) using a Shimadzu^®^ LC system (UFLC-XR, Shimadzu^®^, Kyoto, Japan) coupled to a hybrid triple quadrupole/ion-trap mass spectrometer (API 4000 QTrap, ABSciex^®^, Framingham, MA, USA). Liquid chromatography was performed on a Poroshell 120 EC-C18 column (100  ×  2.1 mm, 2.7 μm, Agilent^®^, Santa Clara, CA, USA) equipped with a guard column (4.0  ×  2.1 mm, 2.7  μm). Injection volume was 5  μL and column temperature 25  °C. A gradient of water (A) and acetonitrile 95% (B) both containing 0.2% of acetic acid were used at a flow rate of 0.2 mL min^−1^ as follows: 0–5 min from 28% to 29% B, 5–15 min from 29% to 30% B, 15–16 min from 30% to 100% B, 16–18 min 100% B, 18–19 min from 100% to 28% B, and re-equilibration with 28% B. The ESI interface was operated using the following parameters: curtain gas 30 psi, temperature: 300 °C, gas1 30 psi; gas2 40 psi, ion spray voltage 5000 V. For detection, the declustering potential was set at 56 V and the entrance potential 10 V. The collision energy was applied at 47 eV for bi-charged ions [M + 2H]^2+^, [M + 2H − H_2_O]^2+^ and at 31 eV for the tri-charged ion [M + 3H − 2H_2_O]^3+^ to give the characteristic product ion at *m*/*z* 327.2, 343.2 or 371.2. Collision cell exit potentials was 20 and 18 V for bi- and tricharged ion respectively.

The following multiple reaction monitoring (MRM) transitions were monitored with the ion source in positive mode: *m*/*z* 1324.2→327.2, 1315.2→327.2 and 877.2→327.2 for ovatoxin-a (OVTX-a); 1346.3→371.2, 1337.3→371.2 and 891.8→327.2 for OVT-b; 1354.3→371.2, 1345.3→371.2 and 897.2→327.2 for OVTX-c; 1332.2→327.2, 1323.2→327.2 and 882.5→327.2 for OVTX-d; 1332.2→343.2, 1323.2→343.2 and 882.5→343.2 for OVTX-e; 1338.3→327.2, 1329.3→327.2 and 886.5→327.2 for OVTX-f; and 1340.2→327.2, 1331.2→327.2 and 887.8→327.2 for palytoxin (PLTX). All toxins were quantified against the palytoxin standard (Wako Chemicals GmbH, Neuss, Germany) assuming similar molar response and expressed as PLTX equivalent (PLTX-eq.). Limit of detection (LOD) and of quantification (LOQ) were 20 and 40 ng PLTX-eq. mL^−1^, respectively.

## Figures and Tables

**Table 1:** Measurements of *Ostreopsis* cf. *ovata* cells (mean, range and number of cells measured) from monoclonal cultures and field samples as obtained from light microscope (at 200× magnification) photomicrographs using an image processing software (AxioVision LE). DV = dorso-ventral length (depth); AP = antero-posterior length (height).

Sample	DV	Wide (W)	DV/W Ratio	AP
Cultures (all)	40.8 (23.7–60.1, *n =* 237)	31 (15.4–48.9, *n =* 203)	1.33 (1.04–1.68, *n =* 238)	28.1 (18.6–44.5, *n =* 26)
LM062	50.3 (34.1–58, *n =* 62)	40.0 (29–48.9, *n =* 57)	1.27 (1.06–1.5, *n =* 62)	37.9 (35.3–44.5, *n =* 4)
LM086	35.5 (27.8–46.3, *n =* 63)	28.8 (22.8–44.7, *n =* 53)	1.25 (1.04–1.44, *n =* 63)	27.3 (23.5–34.2, *n =* 9)
LM129	33.7 (23.7–49.5, *n =* 66)	24.1 (15.4–39.3, *n =* 62)	1.40 (1.05–1.65, *n =* 66)	22.7 (19.7–25.5, *n =* 4)
LM130	45.3 (27.4–60.1, *n =* 46)	32.1 (20.7–40.9, *n =* 31)	1.45 (1.23–1.68, *n =* 47)	27 (18.6–39.4, *n =* 9)
Field	49.9 (29.9–65.9, *n =* 81)	32.6 (17.1–45.9, *n =* 67)	1.53 (1.31–1.79, *n =* 88)	23.4 (16.7–30.7, *n =* 19)
All specimens	43.1 (23.7–65.9, *n =* 318)	31.4 (15.4–48.9, *n =* 270)	1.38 (1.04–1.79, *n =* 326)	26.1 (16.7–44.5, *n =* 45)

**Table 2:** Toxin profile in marine invertebrates (whole tissue homogenates) collected during the 2017 *Ostreopsis* cf. *ovata* bloom in Currais Archipelago, southern Brazil. Sea urchins (*Echinometra lucunter*), sea cucumber (*Holothuria grisea*) and coral (*Palythoa* sp.) were sampled from Currais on February 28th. Mussels (*Perna perna*) were collected on the same date in the nearby location of Galheta Island (where *Ostreopsis* cells were much less abundant), and analyzed either directly after sampling or following a 24-h transplantation (“transp.”) period in Currais. Except for a pool of coral polyps, samples constituted one individual each. Average toxin amounts (“Mean total”) are expressed in µg PLTX-eq. kg^−1^ of the animal whole tissue. LOD = limit of detection; LOQ = limit of quantitation.

Animal	Site	*n*	Mean Total (Min–Max)	%OVT-a (Min–Max)	%OVT-b (Min–Max)	%OVT-c (Min–Max)	%OVT-d (Min–Max)	%OVT-e (Min–Max)
Sea urchin	Currais	4	<LOD	<LOD	<LOD	<LOD	<LOD	<LOD
Sea cucumber	Currais	1	<LOD	<LOD	<LOD	<LOD	<LOD	<LOD
Coral	Currais	1	29.5	67.8%	32.2%	<LOD	<LOD	<LOD
Mussel	Currais	5	22.4 (<LOQ–32.9)	68.4% (65.9–70.4)	31.6% (29.6–34.1)	<LOD	<LOD	<LOD
Mussel	Currais (transp.)	5	98.0 (52.6–131)	68.5% (60.1–77.1)	26.7% (22.9–32.1)	6.18% (2.62–9.75)	<LOD	3.80% (2.85–4.48)

**Table 3:** Intracellular toxin concentrations and toxin profile in *O.* cf. *ovata* strains isolated from selected regions in the Mediterranean and along the Brazilian coast.

Origin	OVTX-a	OVTX-b	OVTX-c	OVTX-d/e	Others ^3^	Total (pg cell^−1^)	Reference
Italy	47–56%	34–37%	4–8%	15–18%	0.5–3%	12.0–20.0	[59]
Italy	~50–70% ^1^	~20–25% ^1^	~0–5% ^1^	~5–25% ^1^	~0–5% ^1^	6.0–15.8	[41]
France	51–61%	14–16%	4–6%	6–18%	5–17%	22.5–300	[7]
Spain	52–59%	20–29%	3–6%	12–16%	0.9–1.6%	50.0–250	[8]
Greece	76.2%	N/A ^2^	N/A ^2^	20.4%	3.4%	44.0	[60]
Brazil (NE)	56–61%	31–37%	0.3–0.7%	3–7%	N/A	21.0–43.4	[61]
Brazil (SE)	19–45%	27–51%	2–18%	3–4–0%	N/A	60.0–468	[62]
Brazil (S)	57–59%	30–35%	1.5–5%	5.5–8%	N/A	11.3–35.5	Present study

^1^ Inferred from graphical data. ^2^ No information about OVTX-b or OVTX-c. ^3^ Isobaric palytoxin, mascarenotoxins, ovatoxin-f and -g, -i, -j, -k.

**Table 4:** Oligonucleotide primers used in the present study.

Primer	Sequence	Reference
ITSfw	5′-GTAGGTGAACCTGCGGAAGG-3′	[74]
FD8	5′-GGATTGGCTCTGAGGGTTGGG- 3′	[75]
D3B	5′-TCGGAGGGAACCAGCTACTA-3′	[74]
OSTD10R	5ʹ-GCACTGAAAATGAAAATCAAGC-3′	[2]
